# 1,4-Di­aza­bicyclo­[2.2.2]octane-1,4-diium bis­(3-chloro­benzoate)

**DOI:** 10.1107/S1600536814000610

**Published:** 2014-01-18

**Authors:** Zi-Shuo Yao, Osamu Sato

**Affiliations:** a Institute for Materials Chemistry and Engineering, Kyushu University, 6-1, Kasuga-koen, Fukuoka, 816-8580, Japan

## Abstract

In the title salt C_6_H_14_N_2_^2+^·2C_7_H_4_ClO_2_^−^, two 3-chloro­benzoate (3CBA) anions are bridged by one diprotonated 1,4-di­aza­bicyclo­[2.2.2]octane-1,4-diium (H_2_DABCO^2+^) dication through N—H⋯O hydrogen bonds. In this way, a trimeric unit is generated, in which the mean planes of the two 3CBA anions are twisted with respect to each other by a dihedral angle of 59.87 (9)°. The trimeric units are linked into a three-dimensional network *via* weak C—H⋯O inter­actions.

## Related literature   

For related studies on co-crystals of DABCO and carb­oxy­lic acids, see: Arman *et al.* (2011 ▶); Skovsgaard & Bond (2009 ▶); Meehan *et al.* (1997 ▶); Rosli *et al.* (2006 ▶); Burchell *et al.* (2001 ▶).
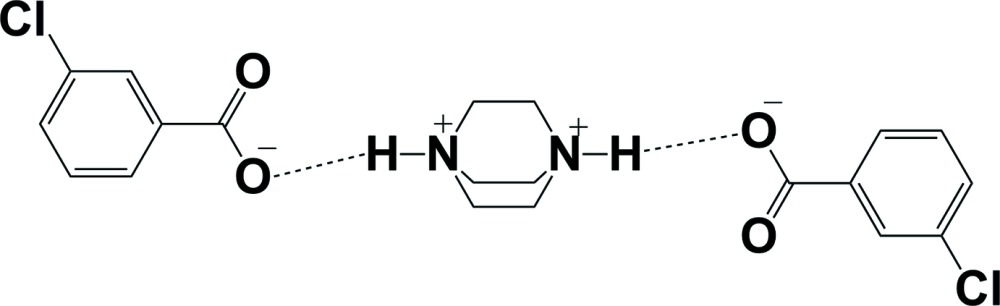


## Experimental   

### 

#### Crystal data  

C_6_H_14_N_2_^2+^·2C_7_H_4_ClO_2_^−^
*M* *_r_* = 425.29
Triclinic, 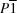

*a* = 7.332 (4) Å
*b* = 10.512 (6) Å
*c* = 13.517 (7) Å
α = 79.74 (3)°
β = 76.68 (2)°
γ = 85.47 (2)°
*V* = 996.8 (9) Å^3^
*Z* = 2
Mo *K*α radiation
μ = 0.36 mm^−1^
*T* = 123 K
0.08 × 0.07 × 0.06 mm

#### Data collection  

Rigaku Saturn70 diffractometer
Absorption correction: multi-scan (*CrystalClear*; Rigaku, 2008 ▶) *T*_min_ = 0.874, *T*_max_ = 1.000
6989 measured reflections
3671 independent reflections
2777 reflections with *I* > 2σ(*I*)
*R*_int_ = 0.034

#### Refinement  

*R*[*F* ^2^ > 2σ(*F* ^2^)] = 0.037
*wR*(*F* ^2^) = 0.097
*S* = 0.91
3671 reflections
253 parameters
H-atom parameters constrained
Δρ_max_ = 0.40 e Å^−3^
Δρ_min_ = −0.33 e Å^−3^

### 

Data collection: *CrystalClear* (Rigaku, 2008 ▶); cell refinement: *CrystalClear*; data reduction: *CrystalClear*; program(s) used to solve structure: *SHELXS97* (Sheldrick, 2008 ▶); program(s) used to refine structure: *SHELXL2013* (Sheldrick, 2008 ▶); molecular graphics: *SHELXTL* (Sheldrick, 2008 ▶); software used to prepare material for publication: *publCIF* (Westrip, 2010 ▶).

## Supplementary Material

Crystal structure: contains datablock(s) I. DOI: 10.1107/S1600536814000610/jj2180sup1.cif

Structure factors: contains datablock(s) I. DOI: 10.1107/S1600536814000610/jj2180Isup2.hkl

Supporting information file. DOI: 10.1107/S1600536814000610/jj2180Isup3.cml

CCDC reference:

Additional supporting information:  crystallographic information; 3D view; checkCIF report

## Figures and Tables

**Table 1:** Hydrogen-bond geometry (Å, °)

*D*—H⋯*A*	*D*—H	H⋯*A*	*D*⋯*A*	*D*—H⋯*A*
N1—H1*N*⋯O3	0.90	1.64	2.536 (2)	178
N2—H2*N*⋯O2	0.90	1.63	2.528 (3)	175
C3—H3⋯O3^i^	0.95	2.59	3.523 (3)	169
C18—H18*B*⋯O4^ii^	0.99	2.43	3.311 (3)	147
C19—H19*B*⋯O1^iii^	0.99	2.56	3.552 (3)	178

Symmetry codes: (i) 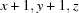; (ii) 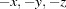; (iii) 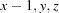.

**Fig. 1.**
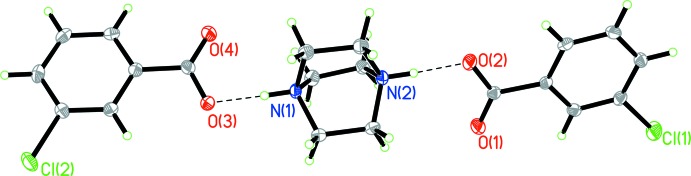
Displacement ellipsoid plot (50% probability level) of the trimer unit. The dashed lines indicate intermolecular N–H···O hydrogen bonds.

**Fig. 2.**
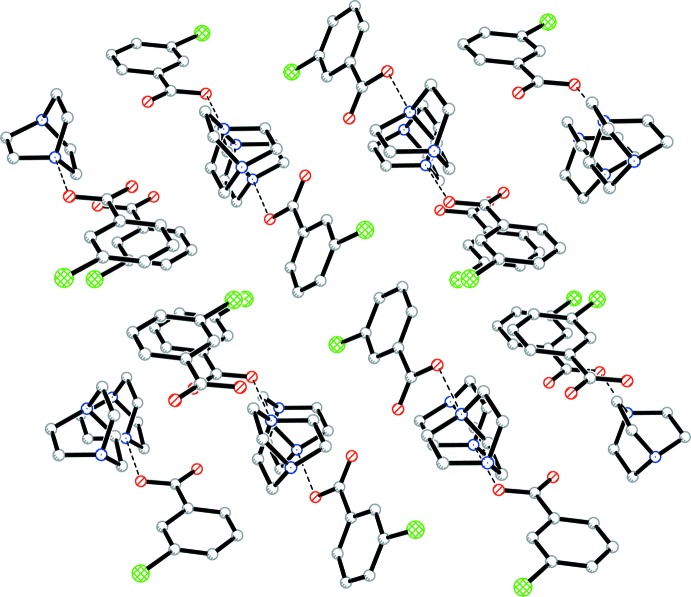
Packing diagram of the title compound viewed along the a axis. The dashed lines indicate intermolecular N–H···O hydrogen bonds forming trimer units. H atoms not involved hydrogen bonding have been omitted for clarity.

## supplementary crystallographic information

### 1. Comment

Molecular based compounds constructed via hydrogen bond interaction can give rise to intriguing properties. Here we report an organic co-crystal of a salt of 1,4-Diazabicyclo[2.2.2]octane (DABCO) and 3-chlorobenzoic acid. The asymmetric unit contains two 3-chlorobenzoate anions and one H_2_DABCO^2+^ cation, where the 3-chlorobenzoate anions are connected by the H_2_DABCO^2+^ cations by strong intermolecular O–H···N hydrogen bond interactions. The short N···O bond lengths (2.528 (3) and 2.536 (2) Å respectively) suggest possible single-well potential curves of the protons in the trimer unit.

### 2. Experimental

Colourless crystals of (1) were isolated from slow evaporation of the acetone solution containing DABCO and 3-chlorobenzoic acid in a mole ratio of 1:2 at room temperature.

### 3. Refinement

Carbon-bound H-atoms were placed in calculated positions (C–H 0.95 and 0.99 Å) and were included in the refinement in the riding model approximation, with Uiso(H) set to 1.2Ueq(C). The N bound H-atoms were located in a difference Fourier map, and were refined with a distance restraints of N–H 0.90±0.01 Å; with Uiso(H) set to 1.2Ueq(N).

### Crystal data

**Table:**

C_6_H_14_N_2_^2+^·2C_7_H_4_ClO_2_^−^	*Z* = 2
*M**_r_* = 425.29	*F*(000) = 444
Triclinic, *P*1	*D*_x_ = 1.417 Mg m^−^^3^
*a* = 7.332 (4) Å	Mo *K*α radiation, λ = 0.71075 Å
*b* = 10.512 (6) Å	Cell parameters from 3510 reflections
*c* = 13.517 (7) Å	θ = 3.1–27.5°
α = 79.74 (3)°	µ = 0.36 mm^−^^1^
β = 76.68 (2)°	*T* = 123 K
γ = 85.47 (2)°	Block, colourless
*V* = 996.8 (9) Å^3^	0.08 × 0.07 × 0.06 mm

### Data collection

**Table:**

Rigaku Saturn70 diffractometer	3671 independent reflections
Radiation source: Rotating Anode	2777 reflections with *I* > 2σ(*I*)
Detector resolution: 28.5714 pixels mm^-1^	*R*_int_ = 0.034
ω scans	θ_max_ = 25.5°, θ_min_ = 3.1°
Absorption correction: multi-scan (*CrystalClear*; Rigaku, 2008)	*h* = −8→8
*T*_min_ = 0.874, *T*_max_ = 1.000	*k* = −12→12
6989 measured reflections	*l* = −16→16

### Refinement

**Table:**

Refinement on *F*^2^	0 restraints
Least-squares matrix: full	Hydrogen site location: inferred from neighbouring sites
*R*[*F*^2^ > 2σ(*F*^2^)] = 0.037	H-atom parameters constrained
*wR*(*F*^2^) = 0.097	*w* = 1/[σ^2^(*F*_o_^2^) + (0.0526*P*)^2^] where *P* = (*F*_o_^2^ + 2*F*_c_^2^)/3
*S* = 0.91	(Δ/σ)_max_ = 0.001
3671 reflections	Δρ_max_ = 0.40 e Å^−^^3^
253 parameters	Δρ_min_ = −0.33 e Å^−^^3^

### Special details

**Table:**

Geometry. All e.s.d.'s (except the e.s.d. in the dihedral angle between two l.s. planes) are estimated using the full covariance matrix. The cell e.s.d.'s are taken into account individually in the estimation of e.s.d.'s in distances, angles and torsion angles; correlations between e.s.d.'s in cell parameters are only used when they are defined by crystal symmetry. An approximate (isotropic) treatment of cell e.s.d.'s is used for estimating e.s.d.'s involving l.s. planes.
Refinement. Refinement of *F*^2^ against ALL reflections. The weighted *R*-factor *wR* and goodness of fit *S* are based on *F*^2^, conventional *R*-factors *R* are based on *F*, with *F* set to zero for negative *F*^2^. The threshold expression of *F*^2^ > σ(*F*^2^) is used only for calculating *R* factors(gt) *etc*. and is not relevant to the choice of reflections for refinement. *R*-factors based on *F*^2^ are statistically about twice as large as those based on *F*, and *R*- factors based on ALL data will be even larger.

### Fractional atomic coordinates and isotropic or equivalent isotropic displacement parameters (Å^2^)

**Table:**

	*x*	*y*	*z*	*U*_iso_*/*U*_eq_	
Cl1	0.85741 (7)	0.29946 (5)	0.56505 (4)	0.02963 (16)	
Cl2	−0.96963 (7)	−0.46471 (5)	0.18722 (4)	0.03041 (16)	
O1	0.33887 (19)	0.09113 (13)	0.42587 (10)	0.0247 (3)	
O2	0.26536 (19)	0.24510 (13)	0.30319 (11)	0.0277 (3)	
O3	−0.40110 (18)	−0.19373 (13)	0.18759 (10)	0.0209 (3)	
O4	−0.22174 (19)	−0.15730 (15)	0.02822 (10)	0.0320 (4)	
N1	−0.1723 (2)	−0.05291 (14)	0.22871 (11)	0.0163 (4)	
H1N	−0.2524	−0.1042	0.2147	0.020*	
N2	0.0517 (2)	0.08989 (15)	0.26718 (11)	0.0170 (4)	
H2N	0.1317	0.1413	0.2812	0.020*	
C1	0.4754 (2)	0.29730 (18)	0.39639 (13)	0.0152 (4)	
C2	0.4815 (3)	0.42300 (18)	0.34315 (14)	0.0183 (4)	
H2	0.3996	0.4506	0.2977	0.022*	
C3	0.6052 (3)	0.50894 (19)	0.35524 (14)	0.0204 (4)	
H3	0.6092	0.5944	0.3175	0.024*	
C4	0.7235 (3)	0.46978 (19)	0.42271 (14)	0.0193 (4)	
H4	0.8105	0.5273	0.4309	0.023*	
C5	0.7124 (2)	0.34535 (19)	0.47776 (14)	0.0178 (4)	
C6	0.5904 (2)	0.25831 (18)	0.46641 (14)	0.0168 (4)	
H6	0.5848	0.1735	0.5054	0.020*	
C7	0.3501 (2)	0.20080 (18)	0.37673 (14)	0.0166 (4)	
C8	−0.4958 (3)	−0.27845 (17)	0.05760 (14)	0.0165 (4)	
C9	−0.6506 (2)	−0.33291 (17)	0.12918 (14)	0.0163 (4)	
H9	−0.6695	−0.3249	0.1998	0.020*	
C10	−0.7755 (3)	−0.39815 (18)	0.09679 (15)	0.0183 (4)	
C11	−0.7508 (3)	−0.41311 (19)	−0.00502 (15)	0.0212 (4)	
H11	−0.8381	−0.4587	−0.0262	0.025*	
C12	−0.5961 (3)	−0.36021 (18)	−0.07544 (15)	0.0213 (4)	
H12	−0.5769	−0.3698	−0.1457	0.026*	
C13	−0.4691 (3)	−0.29346 (18)	−0.04470 (14)	0.0193 (4)	
H13	−0.3633	−0.2579	−0.0938	0.023*	
C14	−0.3593 (3)	−0.20410 (18)	0.09103 (14)	0.0191 (4)	
C15	−0.1980 (3)	0.08017 (18)	0.17522 (15)	0.0211 (4)	
H15A	−0.3308	0.1102	0.1950	0.025*	
H15B	−0.1653	0.0822	0.0997	0.025*	
C16	−0.0712 (3)	0.16953 (18)	0.20515 (15)	0.0210 (4)	
H16A	0.0062	0.2189	0.1423	0.025*	
H16B	−0.1488	0.2319	0.2457	0.025*	
C17	0.0218 (2)	−0.10165 (19)	0.19240 (15)	0.0200 (4)	
H17A	0.0442	−0.1082	0.1184	0.024*	
H17B	0.0414	−0.1890	0.2312	0.024*	
C18	0.1599 (3)	−0.00838 (18)	0.20868 (14)	0.0188 (4)	
H18A	0.2499	−0.0569	0.2474	0.023*	
H18B	0.2315	0.0344	0.1412	0.023*	
C19	−0.2115 (3)	−0.05460 (19)	0.34127 (14)	0.0190 (4)	
H19A	−0.2063	−0.1448	0.3777	0.023*	
H19B	−0.3385	−0.0165	0.3649	0.023*	
C20	−0.0641 (3)	0.02409 (18)	0.36553 (14)	0.0187 (4)	
H20A	−0.1270	0.0892	0.4081	0.022*	
H20B	0.0169	−0.0340	0.4048	0.022*	

### Atomic displacement parameters (Å^2^)

**Table:**

	*U*^11^	*U*^22^	*U*^33^	*U*^12^	*U*^13^	*U*^23^
Cl1	0.0312 (3)	0.0291 (3)	0.0341 (3)	−0.0036 (2)	−0.0212 (2)	−0.0007 (2)
Cl2	0.0256 (3)	0.0346 (3)	0.0307 (3)	−0.0156 (2)	−0.0003 (2)	−0.0061 (2)
O1	0.0320 (8)	0.0181 (8)	0.0259 (8)	−0.0076 (6)	−0.0121 (6)	0.0020 (6)
O2	0.0377 (9)	0.0250 (8)	0.0254 (8)	−0.0111 (6)	−0.0205 (7)	0.0046 (6)
O3	0.0248 (8)	0.0236 (8)	0.0153 (7)	−0.0077 (6)	−0.0037 (6)	−0.0042 (6)
O4	0.0295 (9)	0.0475 (10)	0.0191 (8)	−0.0213 (7)	−0.0021 (6)	−0.0013 (7)
N1	0.0186 (9)	0.0145 (8)	0.0179 (8)	−0.0035 (6)	−0.0077 (7)	−0.0025 (7)
N2	0.0176 (9)	0.0171 (9)	0.0181 (8)	−0.0045 (7)	−0.0074 (7)	−0.0014 (7)
C1	0.0157 (10)	0.0172 (10)	0.0124 (9)	−0.0011 (7)	−0.0011 (7)	−0.0040 (8)
C2	0.0199 (11)	0.0226 (11)	0.0123 (9)	−0.0002 (8)	−0.0043 (8)	−0.0017 (8)
C3	0.0258 (11)	0.0171 (10)	0.0165 (10)	−0.0039 (8)	−0.0021 (8)	0.0000 (8)
C4	0.0186 (10)	0.0217 (11)	0.0181 (10)	−0.0062 (8)	−0.0018 (8)	−0.0047 (8)
C5	0.0159 (10)	0.0228 (11)	0.0160 (10)	−0.0003 (8)	−0.0058 (8)	−0.0040 (8)
C6	0.0172 (10)	0.0169 (10)	0.0153 (9)	−0.0014 (8)	−0.0020 (7)	−0.0018 (8)
C7	0.0156 (10)	0.0203 (11)	0.0139 (10)	−0.0024 (8)	−0.0022 (7)	−0.0037 (8)
C8	0.0186 (10)	0.0126 (10)	0.0183 (10)	0.0009 (7)	−0.0055 (8)	−0.0014 (8)
C9	0.0177 (10)	0.0162 (10)	0.0153 (10)	0.0009 (8)	−0.0030 (8)	−0.0046 (8)
C10	0.0169 (10)	0.0165 (10)	0.0213 (10)	−0.0013 (8)	−0.0037 (8)	−0.0028 (8)
C11	0.0228 (11)	0.0194 (11)	0.0262 (11)	0.0000 (8)	−0.0119 (9)	−0.0080 (9)
C12	0.0272 (12)	0.0227 (11)	0.0158 (10)	0.0016 (8)	−0.0085 (8)	−0.0042 (8)
C13	0.0221 (11)	0.0194 (10)	0.0151 (10)	−0.0003 (8)	−0.0042 (8)	0.0008 (8)
C14	0.0199 (11)	0.0170 (10)	0.0206 (11)	−0.0031 (8)	−0.0064 (8)	0.0003 (8)
C15	0.0228 (11)	0.0183 (10)	0.0232 (11)	−0.0010 (8)	−0.0108 (8)	0.0016 (9)
C16	0.0253 (11)	0.0171 (10)	0.0218 (11)	−0.0016 (8)	−0.0103 (8)	0.0009 (8)
C17	0.0153 (11)	0.0236 (11)	0.0217 (10)	0.0019 (8)	−0.0037 (8)	−0.0068 (9)
C18	0.0165 (10)	0.0212 (11)	0.0184 (10)	−0.0007 (8)	−0.0029 (8)	−0.0034 (8)
C19	0.0203 (11)	0.0216 (11)	0.0145 (10)	−0.0040 (8)	−0.0022 (8)	−0.0017 (8)
C20	0.0225 (11)	0.0203 (10)	0.0135 (10)	−0.0023 (8)	−0.0040 (8)	−0.0020 (8)

### Geometric parameters (Å, º)

**Table:**

Cl1—C5	1.747 (2)	C8—C9	1.397 (3)
Cl2—C10	1.749 (2)	C8—C14	1.508 (3)
O1—C7	1.222 (2)	C9—C10	1.375 (2)
O2—C7	1.290 (2)	C9—H9	0.9500
O3—C14	1.292 (2)	C10—C11	1.382 (3)
O4—C14	1.231 (2)	C11—C12	1.384 (3)
N1—C15	1.475 (2)	C11—H11	0.9500
N1—C19	1.479 (2)	C12—C13	1.385 (2)
N1—C17	1.478 (2)	C12—H12	0.9500
N1—H1N	0.9000	C13—H13	0.9500
N2—C18	1.481 (2)	C15—C16	1.536 (3)
N2—C16	1.481 (2)	C15—H15A	0.9900
N2—C20	1.487 (2)	C15—H15B	0.9900
N2—H2N	0.9000	C16—H16A	0.9900
C1—C2	1.386 (3)	C16—H16B	0.9900
C1—C6	1.396 (3)	C17—C18	1.540 (2)
C1—C7	1.516 (2)	C17—H17A	0.9900
C2—C3	1.384 (3)	C17—H17B	0.9900
C2—H2	0.9500	C18—H18A	0.9900
C3—C4	1.388 (3)	C18—H18B	0.9900
C3—H3	0.9500	C19—C20	1.537 (2)
C4—C5	1.383 (3)	C19—H19A	0.9900
C4—H4	0.9500	C19—H19B	0.9900
C5—C6	1.379 (3)	C20—H20A	0.9900
C6—H6	0.9500	C20—H20B	0.9900
C8—C13	1.387 (3)		
			
C15—N1—C19	109.80 (15)	C13—C12—C11	120.80 (18)
C15—N1—C17	109.77 (15)	C13—C12—H12	119.6
C19—N1—C17	109.86 (14)	C11—C12—H12	119.6
C15—N1—H1N	109.2	C12—C13—C8	120.19 (18)
C19—N1—H1N	109.2	C12—C13—H13	119.9
C17—N1—H1N	109.1	C8—C13—H13	119.9
C18—N2—C16	109.80 (14)	O4—C14—O3	124.69 (17)
C18—N2—C20	109.46 (15)	O4—C14—C8	120.50 (17)
C16—N2—C20	109.79 (15)	O3—C14—C8	114.81 (16)
C18—N2—H2N	109.3	N1—C15—C16	109.14 (15)
C16—N2—H2N	109.3	N1—C15—H15A	109.9
C20—N2—H2N	109.2	C16—C15—H15A	109.9
C2—C1—C6	119.49 (17)	N1—C15—H15B	109.9
C2—C1—C7	120.69 (17)	C16—C15—H15B	109.9
C6—C1—C7	119.79 (17)	H15A—C15—H15B	108.3
C3—C2—C1	121.01 (18)	N2—C16—C15	108.98 (15)
C3—C2—H2	119.5	N2—C16—H16A	109.9
C1—C2—H2	119.5	C15—C16—H16A	109.9
C2—C3—C4	119.77 (18)	N2—C16—H16B	109.9
C2—C3—H3	120.1	C15—C16—H16B	109.9
C4—C3—H3	120.1	H16A—C16—H16B	108.3
C5—C4—C3	118.74 (18)	N1—C17—C18	109.31 (15)
C5—C4—H4	120.6	N1—C17—H17A	109.8
C3—C4—H4	120.6	C18—C17—H17A	109.8
C6—C5—C4	122.26 (18)	N1—C17—H17B	109.8
C6—C5—Cl1	119.69 (15)	C18—C17—H17B	109.8
C4—C5—Cl1	118.05 (14)	H17A—C17—H17B	108.3
C5—C6—C1	118.67 (18)	N2—C18—C17	108.61 (14)
C5—C6—H6	120.7	N2—C18—H18A	110.0
C1—C6—H6	120.7	C17—C18—H18A	110.0
O1—C7—O2	125.20 (17)	N2—C18—H18B	110.0
O1—C7—C1	121.13 (17)	C17—C18—H18B	110.0
O2—C7—C1	113.64 (17)	H18A—C18—H18B	108.3
C13—C8—C9	119.23 (17)	N1—C19—C20	108.85 (14)
C13—C8—C14	120.32 (17)	N1—C19—H19A	109.9
C9—C8—C14	120.45 (16)	C20—C19—H19A	109.9
C10—C9—C8	119.57 (17)	N1—C19—H19B	109.9
C10—C9—H9	120.2	C20—C19—H19B	109.9
C8—C9—H9	120.2	H19A—C19—H19B	108.3
C9—C10—C11	121.69 (18)	N2—C20—C19	109.05 (14)
C9—C10—Cl2	119.01 (15)	N2—C20—H20A	109.9
C11—C10—Cl2	119.29 (14)	C19—C20—H20A	109.9
C10—C11—C12	118.52 (17)	N2—C20—H20B	109.9
C10—C11—H11	120.7	C19—C20—H20B	109.9
C12—C11—H11	120.7	H20A—C20—H20B	108.3
			
C6—C1—C2—C3	2.6 (3)	C9—C8—C13—C12	0.9 (3)
C7—C1—C2—C3	−175.35 (16)	C14—C8—C13—C12	−179.08 (17)
C1—C2—C3—C4	−0.9 (3)	C13—C8—C14—O4	−0.8 (3)
C2—C3—C4—C5	−1.0 (3)	C9—C8—C14—O4	179.18 (18)
C3—C4—C5—C6	1.3 (3)	C13—C8—C14—O3	178.35 (17)
C3—C4—C5—Cl1	−178.13 (14)	C9—C8—C14—O3	−1.6 (3)
C4—C5—C6—C1	0.4 (3)	C19—N1—C15—C16	56.50 (19)
Cl1—C5—C6—C1	179.80 (13)	C17—N1—C15—C16	−64.36 (19)
C2—C1—C6—C5	−2.3 (3)	C18—N2—C16—C15	56.32 (19)
C7—C1—C6—C5	175.65 (15)	C20—N2—C16—C15	−64.07 (18)
C2—C1—C7—O1	−177.41 (18)	N1—C15—C16—N2	7.0 (2)
C6—C1—C7—O1	4.6 (3)	C15—N1—C17—C18	56.14 (19)
C2—C1—C7—O2	4.5 (2)	C19—N1—C17—C18	−64.69 (19)
C6—C1—C7—O2	−173.43 (16)	C16—N2—C18—C17	−64.37 (19)
C13—C8—C9—C10	−1.3 (3)	C20—N2—C18—C17	56.22 (19)
C14—C8—C9—C10	178.71 (17)	N1—C17—C18—N2	7.0 (2)
C8—C9—C10—C11	0.9 (3)	C15—N1—C19—C20	−65.01 (18)
C8—C9—C10—Cl2	−179.50 (14)	C17—N1—C19—C20	55.80 (19)
C9—C10—C11—C12	−0.2 (3)	C18—N2—C20—C19	−65.10 (19)
Cl2—C10—C11—C12	−179.78 (15)	C16—N2—C20—C19	55.49 (19)
C10—C11—C12—C13	−0.2 (3)	N1—C19—C20—N2	7.6 (2)
C11—C12—C13—C8	−0.2 (3)		

### Hydrogen-bond geometry (Å, º)

**Table:**

*D*—H···*A*	*D*—H	H···*A*	*D*···*A*	*D*—H···*A*
N1—H1*N*···O3	0.90	1.64	2.536 (2)	178
N2—H2*N*···O2	0.90	1.63	2.528 (3)	175
C3—H3···O3^i^	0.95	2.59	3.523 (3)	169
C18—H18*B*···O4^ii^	0.99	2.43	3.311 (3)	147
C19—H19*B*···O1^iii^	0.99	2.56	3.552 (3)	178

Symmetry codes: (i) *x*+1, *y*+1, *z*; (ii) −*x*, −*y*, −*z*; (iii) *x*−1, *y*, *z*.
